# Beliefs and practices during pregnancy and childbirth in urban slums of Dhaka, Bangladesh

**DOI:** 10.1186/1471-2458-12-791

**Published:** 2012-09-17

**Authors:** Nuzhat Choudhury, Allisyn C Moran, M Ashraful Alam, Karar Zunaid Ahsan, Sabina F Rashid, Peter Kim Streatfield

**Affiliations:** 1 Research and Evaluation Division, BRAC, Dhaka, 1212, Bangladesh; 2 International Centre for Diarrhoeal Disease Research, Bangladesh (icddr,b), Dhaka, 1212, Bangladesh; 3 Department of International Health, Johns Hopkins Bloomberg School of Public Health, Baltimore, MD, USA; 4 Sydney School of Public Health, The University of Sydney, Sydney, Australia; 5 South Asian Human Development Sector, World Bank, Dhaka, Bangladesh; 6 James P. Grant School of Public Health, BRAC University, Dhaka, Bangladesh

**Keywords:** Beliefs and practices, Maternal care, Urban-slum, Bangladesh

## Abstract

**Background:**

Worldwide urbanization has become a crucial issue in recent years. Bangladesh, one of the poorest and most densely-populated countries in the world, has been facing rapid urbanization. In urban areas, maternal indicators are generally worse in the slums than in the urban non-slum areas. The *Manoshi* program at BRAC, a non governmental organization, works to improve maternal, newborn, and child health in the urban slums of Bangladesh. This paper describes maternal related beliefs and practices in the urban slums of Dhaka and provides baseline information for the *Manoshi* program.

**Methods:**

This is a descriptive study where data were collected using both quantitative and qualitative methods. The respondents for the quantitative methods, through a baseline survey using a probability sample, were mothers with infants (n = 672) living in the *Manoshi* program areas. Apart from this, as part of a formative research, thirty six in-depth semi-structured interviews were conducted during the same period from two of the above *Manoshi* program areas among currently pregnant women who had also previously given births (n = 18); and recently delivered women (n = 18).

**Results:**

The baseline survey revealed that one quarter of the recently delivered women received at least four antenatal care visits and 24 percent women received at least one postnatal care visit. Eighty-five percent of deliveries took place at home and 58 percent of the deliveries were assisted by untrained traditional birth attendants. The women mostly relied on their landladies for information and support. Members of the slum community mainly used cheap, easily accessible and available informal sectors for seeking care. Cultural beliefs and practices also reinforced this behavior, including home delivery without skilled assistance.

**Conclusions:**

Behavioral change messages are needed to increase the numbers of antenatal and postnatal care visits, improve birth preparedness, and encourage skilled attendance at delivery. Programs in the urban slum areas should also consider interventions to improve social support for key influential persons in the community, particularly landladies who serve as advisors and decision-makers.

## Background

Global urbanization has become a crucial issue in recent years. The urban population is expected to increase by 84 percent, from 3.4 billion in 2009 to 6.3 billion in 2050 [1]. Bangladesh, along with other Asian countries, has experienced rapid urban growth in the recent decades [2,3]. This rapid urbanization in Bangladesh, coupled with the growth of urban slums, is likely to have profound implications on its health profile, especially on maternal and child health [2]. Maternal and child health is strongly associated with beliefs and practices around pregnancy and childbirth which has implications for the health of the child and mother after the birth.

The maternal mortality ratio (MMR) in Bangladesh was 320 per 100,000 live births in 2001 which decreased to 194 per 100, 000 live births in 2010 [4,5]. Bangladesh is presently on track to achieve the primary target of MDG −5 with a goal to reduce maternal mortality. Despite this achievement the condition in urban slums is worse compared to urban non-slum areas with respect to antenatal care consisting of a medically trained provider (62% vs. 85%), delivery at a facility (12% vs. 46%), and skilled assistance at delivery (18% vs. 56%) respectively [2]. This makes urban health issues, especially of the slum dwellers, a high priority. It is therefore crucial to address maternal health of the urban slum dwellers in Bangladesh. Usually in urban slums, the maternal services are offered at home or in static service delivery sites operated by non-governmental organization (NGO) field workers. In some instances, services are available at clinics or dispensaries managed by NGOs, the government or the private sector [2].

Keeping this in mind, BRAC (formerly known as the Bangladesh Rural Advancement Committee), a large indigenous NGO, initiated a comprehensive Maternal, Newborn and Child Health (MNCH) program in the urban slums, known as *Manoshi*, in 2007 [6]. The program aims to reduce childbirth and pregnancy related morbidity and mortality among women, newborns, and children in the urban slums of Bangladesh [6,7]. During the inception phase of *Manoshi*, a baseline survey and a qualitative research study were conducted in Dhaka city slums [6,8]. As a part of formative research, the qualitative data collection explored perceptions around maternal care while the quantitative study provided baseline information on key indicators. Other large scale national surveys, such as the Demographic and Health Surveys do not disaggregate data by urban slum and non-slum areas. However, the findings of this paper will be used to compare the key indicators with the endline data to assess the program effectiveness and to garner support for the expansion of such programs. The objective of this paper is to describe baseline information on different aspects of maternal care. This paper is the second of its kind in a part of two papers on the *Manoshi* program on care practices. The first paper outlined the newborn care practices and was published elsewhere [9].

## Methods

This study utilized both quantitative and qualitative methods [8,9]. The quantitative and qualitative parts were carried out concurrently but independently. The quantitative component employed a cross sectional baseline survey in the six *Manoshi* program slum areas (*Gulshan, Shyampur, Kamrangir Char, Shabujbag, Mohammadpur, and Uttara*) between August and October 2007. The sampling strategy followed a two-stage random cluster design. Each cluster was comprised of 175 households (an identifiable segment of a patty/block). Fifty clusters from each area were selected by probability proportional to the size in terms of the slum population. In the second stage, from each cluster, around twelve women with an infant and currently living in the same slum were randomly selected (n = 672). The qualitative component explored beliefs and practices around maternal care. Thirty six in-depth semi-structured interviews were conducted in two slums in Dhaka city between March and October 2007 (*Korail* and *Kamrangir Char)*. The *Korail* slum is one of the biggest slums in the centre of Dhaka metropolitan area with a population of about 30,000; whereas, *Kamrangir Char* is in the southern part of Dhaka city and has a population of about 98,000. A total of 18 currently pregnant women who have had previous live births and 18 women with a recent delivery but who were not currently pregnant were randomly selected from program records from these two slum areas to assess the perceptions and beliefs during pregnancy and the postnatal period. The study was approved by the BRAC internal review committees and icddr,b Institutional Review Board (IRB). All participants gave informed consent prior to completing the quantitative survey and qualitative interviews.

### Data collection and analysis

For the baseline survey, trained interviewers collected information on reproductive history, knowledge and perception on pregnancy, delivery, and post-partum care. The survey data were analyzed using SPSS v 13 and Stata v 9.1. For the qualitative component, three interviewers trained in social science interviewed the respondents. Beliefs and practices relating to various aspects of maternal care were explored by engaging in conversations with the respondents using a semi-structured interview guide. The topics of discussion included: perceptions and practices pertaining to antenatal care, postnatal care, and delivery care, care practices during pregnancy, birth preparedness as well as the persons influencing maternal care decisions. The research team reviewed the transcripts to develop a code list for the topics related to the research questions. Codes were applied manually to the transcripts by the interviewers [10]. The text pertaining to the codes were organized in a matrix and translated into English.

## Results

The background characteristics of respondents in the baseline survey are presented in Table 1. Ninety-three percent of the respondents were married before reaching the age of 20, having a median age of 16 years during marriage. The majority of the respondents (81%) were between 15 and 29 years. Half of the respondents reported as receiving education either from formal or religious schools. More than half of the respondents (52%) were living in the slum for two years or less at the time of the interview. Regular exposure to the electronic media, such as the television, was widespread among the respondents; 86 percent of respondents reported watching television at least once a week.

**Table 1:** Baseline characteristics of the sample from the cross sectional survey

**Characteristics**	** n**	** %**
Electricity in household	601	89.4
Piped drinking water in household	376	56
Ever attended school/madrassa	361	53.7
Religion (Islam)	663	98.7
Marital status (currently married)	663	98.7
Currently employed	116	17.3
Number of children ever born		
1–2	396	58.9
3–4	189	28.1
5 or more	87	13.0
Total	N=672	100.0

### Antenatal care

Findings from the baseline survey revealed that three of every four women (75%) had received at least one antenatal care visit for the most recent birth, with 27 percent of women having reported as receiving four or more antenatal care visits (Table 2). In-depth interviews suggested that most of the women attended antenatal care to confirm pregnancy either via urine test or physical examination. Some women reported as not routinely seeking antenatal care at facilities as they did not see an urgent need for it, except to reconfirm the pregnancy.

**Table 2:** Percent distribution of women who had a live birth in the last one year by antenatal care, delivery care and postnatal care for the most recent birth

	**n**	**%**	**CI**
Received any antenatal care during the last pregnancy	507	75.4	72.0-78.5
Received 4+ antenatal care during the last pregnancy	179	26.6	23.2-29.9
Place of last antenatal care visit			
No antenatal care visit	165	24.6	21.2-27.8
Home	16	2.4	1.2-3.5
Birthing Hut	37	5.5	3.7-7.2
Government hospital	101	15.0	12.3-17.7
NGO	271	40.3	36.6-44.0
Pharmacy/Chamber/Private clinic	82	12.2	9.7-14.6
Services received during antenatal care visit*			
Height/weight measured	293	43.6	39.8-47.3
Blood pressure measured	183	27.2	24.0-30.7
Blood tested	77	11.5	9.2-14.0
Urine tested	121	18.0	15.2-21.0
Abdomen examined	409	60.9	57.1-64.4
Internal examination	5	.7	0.3-1.7
Ultrasonic test	95	14.1	11.7-16.9
Tetanus vaccination	61	9.1	7.1-11.4
Iron supplementation	78	11.6	9.4-14.2
Place of delivery, Home	567	84.4	81.1-86.7
Assistance during delivery			
Self	6	.9	0.1-1.6
Relative/Neighbour	42	6.3	4.4-8.0
Untrained TBA	390	58.0	54.2-61.7
Trained TBA	135	20.1	17.0-23.1
BRAC health worker	8	1.2	0.3-2.0
Midwife/Family Welfare Visitor	40	5.9	4.1-7.7
MBBS doctor	51	7.6	5.5-9.5
Received any postnatal care during the last pregnancy	160	23.8	20.7-27.1
Total	N=672	100%	

*Multiple response.

### Birth preparedness

In the *Manoshi* program, birth preparedness included selecting a skilled birth attendant, arranging articles needed for safe birth, identifying where to go in case of emergencies, and arranging money and transport during pregnancy. In the in-depth interviews, the respondents reported having almost no concept of birth preparedness. Many were fatalistic in attitude and placed their trust and faith in Allah in the event of an emergency.

"‘We are poor people, Allah will help us; He will never give us any burden which goes beyond our capacity’ (Pregnant woman, 26 years old)."

"‘I did not have any plan; I knew that in the event of the delivery pain, I would be able to call any of the neighbours who would bring a ‘dai’ (Traditional birth attendant) for delivery’ (Recently delivered women, 23 years old)."

The majority of respondents reported as not saving money in case of an emergency, citing that they would get a loan or borrow money from their landlady if needed. The women also stated as relying on their landladies for advice and suggestions during pregnancy.

### Birth attendants and privacy

In the baseline survey, 84 percent of women reported giving birth at home assisted by traditional birth attendants (TBA) (Table 2). In the in-depth interviews, women reported widespread preference for TBAs. TBAs were low-cost and women preferred to receive care in the home, unless there was a perceived complication.

"‘Dai is less expensive, they are from our same locality then why should I go to hospital for delivery’ (Recently delivered woman, 22 years old)."

Women reported as having a preference for giving birth in their natal home, to ensure care and comfort from their families. However, this was not often feasible, and women often gave birth in the slums. Due to space limitations in the slum setting, each dwelling is usually not more than one room. During delivery, male and younger family members typically had left the dwelling to give privacy to the delivering woman. However, women reported that it was difficult to retain this privacy after the delivery, as family members needed to return.

### Postnatal care

In this study, postnatal care is defined as a visit to a health facility or a health worker coming to the house to check on the woman and her baby after the birth. In the baseline survey, about one quarter (24%) of the women reported receiving postnatal care, with five percent reported four or more postnatal care visits. In the in-depth interviews, women reported remaining on the floor, usually on a mat after the birth of the baby until the TBA cut the umbilical cord and delivered the placenta. The woman was then washed, especially the lower part of the body, by the TBA or close relatives such as the mother or sister-in-law. The TBA usually helped the woman for the first day after birth; then the woman relied on family members and/or her landlady. Sixteen out of 18 women who had recently delivered reported that they did not receive any postnatal care. These women reported not seeking care because they did not perceive any major health problems. Minor headaches and body aches were part of the delivery procedure which did not create demand for seeking postnatal care.

### Restrictions during post-partum period

In-depth interviews revealed that women’s diets were rigidly controlled after birth. Women were advised to eat dry food which was cooked without water, and rice with mashed potato and black cumin seed. These foods were believed to keep the stomach of a woman cool and initiate the production of breast milk. Most of the women in the in-depth interviews mentioned that after giving birth, the flesh inside became flaccid and soft and therefore, the mother must avoid ‘hard’ foods. Women reported that family members, primarily elderly women or landladies advocated for traditional practices with regard to food restrictions.

"“It is good to follow the elderly women in taking food especially after the delivery, this restriction at least will not be harmful for me” (A recently delivered woman, 19 years old)."

All the women reported that they were aware that they should not do heavy work for up to 40 days after delivery. But in reality, this varied depending on support within the household and place of delivery. Many women reported initiating normal activity within 10 to 12 days after delivery.

### Influential people in the slum

In the in-depth interviews, the landlady emerged as an influential figure. Landladies typically owned large compounds within the slum and rented out single rooms. Slum households are typically comprised of nuclear families, with extended family members remaining in native rural areas. Women reported relying on their landlady for pregnancy and delivery-related decisions, and for credits/loans if needed.

"“I know a local dai, but do not contact her unless my Bariwali (landlady) introduces me to the local dai. Bariwali is very helpful, in case of emergency I can borrow money from her because she has her own money and can manage giving money without asking her husband” (Pregnant woman, 26 years old)."

Landladies were perceived to be socially empowered, as they had resided in the slum for long periods of time and had knowledge of reliable and available TBAs to assist with birth. In fact, the landlady’s name was widely used to identify the respondent’s household.

## Discussion

There is a difference in maternal health indicators for urban slum areas when compared with the urban non slum areas. It is therefore essential to understand maternal health behaviours and practices in an effort to improve maternal health indicators. This study combines both quantitative and qualitative methods to understand the maternal care practices to inform the comprehensive MNCH program- *Manoshi*.

Findings from this study are similar to maternal practices and behaviours in the rural areas of Bangladesh [11-16]. Although there is increased access to health services in urban areas (including pharmacies, and private and public health facilities), women in this study maintained practices and behaviours similar to those in the rural areas. This may be due to the fact that these women had only been living in the urban slum on an average of two years and that these behaviours have been deeply ingrained, culturally accepted, and difficult to change over the years. It is believed that cultural concerns in South Asia could be important barriers for seeking antenatal care [12,13]. Other research findings from South Asia and Africa suggest that community health workers (CHWs) and women’s group influence care-seeking practices [11,16-18]. Despite common cultural barriers, women in other studies are more likely to seek antenatal care after a home visit during pregnancy by a CHW [18]. In the Hala trial in Pakistan, women with at least one home visit by a CHW during pregnancy reported increases in use of antenatal care and facility-based delivery [17]. Behaviour change, however, may take time and interventions should run for an appropriate length of time to promote consistent information [19]. Several studies have indicated that training and supervision of CHWs are critical for successful implementation [20]. Thus, training CHWs should be a key strategy for the *Manoshi* program to improve the demand for routine care, promoting birth preparedness, and the uptake of the recommended number of antenatal care and postnatal care visits.

Key findings of this study point to issues with regard to privacy and lack of space during delivery. In South Asia including Bangladesh, the mother and the child are usually isolated immediately after delivery due to beliefs about pollution and impurity linked to the delivery process. In addition, after delivery, the mother and baby are considered to be in a vulnerable state [13,21-23]. Confinement of the mother and baby is believed to protect them from exposure to disease and evil spirits. The period of seclusion and confinement varies across regions [13,24]. In many regions, the confinement period can last up to 40 days. In this study, women were found to have desired privacy during delivery as well as after the birth, but could not maintain this practice due to space constraints. The *Manoshi* program has initiated a ‘delivery centre’ in each slum to ensure access to clean delivery assisted by trained TBAs as well as privacy during delivery and after birth. This approach will be evaluated at the end of the program to determine acceptability, feasibility, and cost-effectiveness as a strategy to expand to other urban slum settings.

Social support is an important element for pregnancy and delivery. A recent study summarising a number of *Manoshi* formative research studies demonstrated strong social networks for responding to social and economic problems, but less effective social networks for health related problems [6]. In rural areas, female family members were found to provide extensive support during pregnancy, delivery and in the care of the mother and newborn after birth. In the present study we found that slum women often live in nuclear families, often at great distances from their female family members and that is why women relied heavily on their landladies for support. Landladies played a significant supportive role in terms of helping women with loans and/or credit, participating in childbirth, and helping the woman and child during the postpartum period. In future programs, they could be targeted for health education messages. In addition, CHW, such as BRAC’s *Shasthya Shebika* could provide social support in future programs. As this study was conducted during *Manoshi’s* inception phase, these workers were not yet perceived to be influential.

There are some limitations that need to be considered when interpreting the findings. The findings are based on self-reported maternal care practices, and may therefore differ from actual practices. In addition, we did not include primi gravidas in the qualitative part and were unable to capture their beliefs and perceptions. The baseline survey included women with children under the age of one year who might not have delivered in the study area and therefore the results may not reflect behaviours of all women in the slums of this study. However, given the consistency of findings in both the quantitative survey and qualitative interviews, we are confident that the findings represent actual practices and are representative of the urban slum maternal care practices.

Our findings on maternal care behaviours and practices have implications for the design of slum-based maternal and neonatal child health programs for *Manoshi.* Our research findings are quite similar with Urban Health Survey 2006 for most of the maternal health indicators [2]. This study demonstrated that although maternal practices are similar in the urban slum and rural areas, women have less social support in urban slums. Women received assistance from traditional birth attendants and/or landladies during delivery. Programs in urban areas, especially in urban slums, should consider interventions to include training and behavioral change messages for women like landladies to ensure proper maternal care throughout the pregnancy, delivery and postpartum periods.

## Conclusions

Behavioral change interventions to increase the numbers of antennal care and postnatal care visits, knowledge of birth preparedness, and ensuring skilled care and privacy at delivery are needed. Programs in urban slum areas should also consider interventions to improve support for women during this vulnerable period, particularly landladies who provide financial and social support. These interventions may improve maternal care practices in urban slum settings.

## Competing interests

The authors declare that they have no competing interests.

## Authors’ contributions

NC, ACM, MAA and SFR conceived of the qualitative study, participated in its design, coordination, data collection, and data analysis. ZAK and PKS participated in design of the quantitative study, coordination, data collection and data analysis. NC, ACM and MAA drafted and finalized the manuscript. ACM was also involved in editing of the manuscript. All authors read and approved the final manuscript.

## Pre-publication history

The pre-publication history for this paper can be accessed here:

http://www.biomedcentral.com/1471-2458/12/791/prepub
